# Hospital Meetings

**Published:** 1901-05-18

**Authors:** 


### HOSPITAL MEETINGS.

### THE LIVERPOOL SCHOOL OF TROPICAL DISEASES.

An important meeting of the committee of the Liverpool School of Tropical Medicine was held recently, when the following members of the committee were present:—Messrs. Alfred L. Jones (chairman), W. Adamson (vice-chairman), Chas. Booth, jun., Professor Boyce, Geo. Brocklehurst, Dr. E. Adam, Dr. Carter, Joseph Chadwick, Col. James Goffey, Major Ronald Ross, E. Johnston, J. O. S. Trafford, and A. H. Milne (hon. sec.)

Since the last meeting of the committee steps had been taken to raise a suitable memorial to commemorate the premature loss of Dr. Walter Myers, who lost his life in Para from yellow fever, whilst investigating that disease, on a mission of the Liverpool School of Tropical Medicine, on January 20th, 1901. The committee have already offered to erect in University College, Liverpool, and University College, Birmingham, memorial brasses, placing on record the loss sustained by the scientific world in the death of Dr. Myers. These offers have been willingly accepted, and in addition a tombstone of Aberdeen granite has been erected over the grave of Dr. Myers, in Para, by the school. It was felt, however, that the devotion of Dr. Walter Myers to the cause of tropical medicine required a more useful form of recognition, and it was consequently resolved to found a perpetual chair in the Liverpool School of Tropical Medicine to be called the Walter Myers chair of tropical medicine, and also to found a supplementary fellowship for the next five years in memory of Dr. Myers to be called the Walter Myers Fellowship of Tropical Medicine. The co-operation of those specially interested in the school was solicited, and met with the most generous response. With the assistance of several leading shipowners in Liverpool, Mr. George Myers and Mr. Charles Samuel (relatives of Dr. Walter Myers), Mrs. George Holt, Mr. James Coats, jun., of Paisley, and others, both the Chair and Fellowship have now been founded. The movement has met with much encouragement from the scientific world, and has had the special assistance of the co-operation of Lord Lister.

At the recent meeting of the Committee the appointments to the Chair and Fellowship were made, viz., to the Walter Myers Chair of Tropical Medicine, Major Ronald Ross, M.D., D.P.H., late I.M.S., and to the Fellowship, Dr. Dutton, M.B., Vict. Major Ross's services to the cause of research in tropical medicine are too well known to require recapitulation, and it is sufficient to point to his work in connection with the mosquito theory of malaria. The value of this is shown by the recent nomination of Major Ross for election to a Fellowship of the Royal Society. Dr. Dutton, who has now been elected to the Walter Myers Fellowship of Tropical Medicine, was elected to the Holt Fellowship of Pathology in 1897. In 1900 he took part in the third malarial expedition of the school to West Africa, which visited Nigeria, and spent over six months there investigating tropical diseases. He has also written papers on malaria and filaria. Since the return of the expedition Dr. Dutton has been engaged in working for the school.

### NATIONAL SANATORIUM FOR CONSUMPTION, BOURNEMOUTH (OPEN-AIR TREATMENT).

The annual general meeting of Governors of this charitable and philanthropic institution was held in London on Thursday, the 9th inst., at the Westminster Palace Hotel, the President, the Right Hon. the Earl of Eldon, occupying the chair. There were also present the Right Hon. Lord Eustace Cecil, M.P., Arthur F. Jeffreys, Esq., M.P., Lieut.-Col. Lewin, J. Gregory White, Esq., M.D., H. E. Acklom, Esq., the Rev. Oldfeld K. Prescot, &c.

The annual report of the committee for the year 1900 was placed before the Governors, the statement of accounts showing a balance in hand of £122. Special measures were considered for meeting the increase in necessary annual expenses incurred by the efficient carrying on of the open-air treatment of patients, which had been successfully adopted for some time past. Measures having been also discussed for obtaining increased funds to carry out the proposed extension of the building, it was resolved that a further appeal for donations and subscriptions should be made to the public with the concurrence and signature of the Earl of Eldon as President.

The following resolution was also unanimously passed:—"That the humble petition of the Governors and Committee of this Charity be placed before His Majesty the King for His gracious continuance of the Royal patronage formerly granted by Her late Majesty Queen Victoria since the year 1869."

### SAMARITAN FREE HOSPITAL.

THE annual meeting of the Samaritan Free Hospital was held on Tuesday, 14th inst., the Right Hon. the Lord LEIGH in the chair. The report showed that the year 1900 was a very successful one, both as regards the medical and surgical work done in the hospital, and the state of the finances. The number of in-patients during the year was: Medical, 277; surgical, 206; total, 483. The total number of out-patients was 7,872.

The annual subscriptions amounted to £1,524, as against £1,534 in 1899. The donations were £3,610, being £1,578 above those received in 1899; whilst the sum of £36, collected in the almsboxes, was an appreciable increase upon that of any former year. The Prince of Wales's Fund gave the usual annual grant of £500 and an additional donation of £50. The Hospital Sunday Fund contributed £383, and the Hospital Saturday Fund £113. The legacies amounted to £376 only.

The Physicians' report stated that very excellent results had been obtained during the year; the cases were, and would continue to be, of a more serious nature than hitherto; not in the matter of disease, but in the methods of operative treatment on the medical side, thus necessitating a longer stay in the hospital. Many cases that before were looked upon as hopeless now received permanent benefit, or were cured by operation. This was especially the case with cancer, for which vaginal hysterectomy had been performed a large number of times during the year without a single death. On the medical side, 158 operations had been performed without a death; three deaths had occurred in the physicians' wards, but these were due to complications or specific disease. By such increasing operative vaginal work the physicians in no way clashed with the surgeons, certain cases only being suitable for each. The surgical report dealt chiefly with the advantages of the private ward system for dangerous operations; it was in all cases beneficial. The out-patient staff, medical and surgical, again called attention to the necessary building reconstruction; the committee were waiting till the termination of a lease of the adjoining cottages. The report and balance-sheet having been adopted, certain alterations in the rules were agreed upon, and Mr. J. R. Diggle's motion that no member of the committee be eligible for re-election who has failed to attend at least three committee meetings in the course of the year, was replaced by an amendment to the contrary effect. Thanks to Lord Leigh ended the meeting.

